# Functional Trajectory and Quality of Life Divergence Following Surgical Versus Conservative Management of Achilles Tendon Contracture in Monozygotic Twins with Duchenne Muscular Dystrophy: A Case Report

**DOI:** 10.3390/children13080988

**Published:** 2026-07-25

**Authors:** Taekyung Lee, Jihyun Kwon, Yeonsu Oh, Han Eol Cho, Dong-wook Rha, Juntaek Hong

**Affiliations:** 1 Department and Research Institute of Rehabilitation Medicine, Severance Hospital, Yonsei University College of Medicine, Seoul 03722, Republic of Korea; sbzld89@yuhs.ac (T.L.); ysysysysys98@yuhs.ac (Y.O.); medicus@yuhs.ac (D.-w.R.); 2 Department of Rehabilitation Medicine, Gangnam Severance Hospital, Rehabilitation Institute of Neuromuscular Disease, Yonsei University College of Medicine, Seoul 06273, Republic of Korea; jihyunkwon95@yuhs.ac (J.K.); wtzephyr@yuhs.ac (H.E.C.)

**Keywords:** Achilles tendon contracture, monozygotic twins, Duchenne muscular dystrophy, quality of life, gross motor function

## Abstract

**Highlights:**

**What are the main findings?**

- Achilles tendon lengthening coincided with attenuated longitudinal motor decline in this twin pair.
- Quality-of-life outcomes were mixed and exploratory across different dimensions.

**What are the implications of the main findings?**

- Evaluation of orthopedic interventions may benefit from looking beyond immediate gait metrics toward broader functional trends.
- Potential long-term functional benefits appear conditional on coupling timely surgery with structured, continuous rehabilitative care.

**Abstract:**

**Background**: The timing and efficacy of Achilles tendon lengthening (ATL) in Duchenne muscular dystrophy (DMD) remain controversial because indiscriminate surgery can accelerate ambulation loss. **Case Description**: This report presents a 5-year longitudinal comparative analysis (2021–2026) of monozygotic twins with identical genetic backgrounds (DMD exon 30–43 deletion) who received comparable rehabilitation and pharmacological management, including concurrent gene therapy in 2025. Twin A was managed conservatively with orthosis and subsequent serial casting for progressive equinus contracture, whereas Twin B underwent early bilateral ATL during transition to the non-ambulatory phase. Despite a temporary postoperative decline, Twin B demonstrated a more stable longitudinal motor trajectory, outperforming Twin A in gross motor function (Gross Motor Function Measure-88: 38.60% vs. 32.85% in 2026) by preserving residual standing and crawling dimensions. Longitudinal KIDSCREEN-52 assessments revealed a clinically meaningful improvement in psychological well-being in Twin B (+10.9 points) relative to Twin A. **Conclusions**: This single-pair twin case serves as a hypothesis-generating observation, highlighting a potential longitudinal association between early surgery and attenuated progressive motor decline within a multi-disciplinary care regimen. Given highly variable psychosocial outcomes, these trends cannot be directly attributed to surgery alone. When evaluating such orthopedic interventions, clinical decisions may benefit from looking beyond immediate gait metrics toward broader, long-term functional trends, though the relative contributions of concurrent therapies remain to be elucidated.

## 1. Introduction

Duchenne muscular dystrophy (DMD) is an X-linked recessive inherited disorder characterized by a progressive deficiency of the dystrophin protein, which leads to unremitting muscle wasting and asymmetric joint contractures [1]. Among these musculoskeletal manifestations, Achilles tendon contracture of the ankle joint is a common complication that significantly compromises ambulatory function, alters gait biomechanics, and elevates the risk of falls [2].

According to Birnkrant et al. [3] and Skalsky et al. [4], performing Achilles tendon lengthening (ATL) indiscriminately in patients with DMD during the ambulatory phase can be counterproductive, as it may paradoxically accelerate the loss of independent ambulation if proximal muscle strength in the hips and knees is insufficient to support the altered biomechanical alignment. Consequently, the definitive indications and efficacy of surgical intervention remain controversial and patient-specific. Clinical consensus dictates that a meticulous, patient-specific approach is warranted—one that carefully balances critical variables, such as optimal surgical timing, preoperative lower extremity muscle strength, and the availability of rigorous postoperative rehabilitation and long-term orthotic management.

This case offers a rare opportunity to explore comparative analysis of monozygotic twins sharing an identical genetic background who followed divergent therapeutic pathways: Twin A was managed strictly with conservative treatment, whereas Twin B underwent early bilateral ATL during transition into the non-ambulatory phase. Through a retrospective evaluation of their longitudinal clinical courses, objective functional metrics, and subjective quality of life (QoL) outcomes over a 5-year follow-up period (May 2021–May 2026), this case report provides exploratory insights regarding the potential role of orthopedic interventions in the comprehensive management of DMD.

## 2. Case Description

### 2.1. Patient Baseline and Diagnostic Workup

The patients were monozygotic male twins who presented with divergent initial functional profiles despite having identical genetic backgrounds. Twin B presented with a noticeable toe-walking gait at 7 years of age and subsequently lost his capacity for independent ambulation by age 8. At that time, he was evaluated at the orthopedic outpatient clinic, where next-generation sequencing (NGS) identified a hemizygous deletion of exons 30–43 in the *DMD* gene. Whole-spine magnetic resonance imaging revealed diffuse fatty infiltration across multiple lower-extremity muscle groups, suggesting a muscular dystrophy, and a subsequent muscle biopsy demonstrated classic myopathic changes, including marked fiber size variation, fiber splitting, and prominent fatty ingrowth, establishing the definitive diagnosis of DMD. Conversely, Twin A exhibited more favorable baseline gross motor function; although he began demonstrating Gowers’ sign at approximately the same age, he maintained the ability to ambulate independently for approximately 5 min at age 8. Following his brother’s definitive diagnosis, Twin A visited our department at the age of 8 years for a formal diagnostic workup. Given their monozygotic status and identical clinical presentation, Twin A’s diagnosis of DMD was confirmed solely via NGS, thereby avoiding an unnecessary, invasive muscle biopsy. The overall chronological timelines for both twins are outlined in Table 1.

### 2.2. Clinical Trajectory and Interventions (2021–2026)

Both patients adhered to identical chronological schedules for all inpatient admissions and outpatient visits. Over the course of their management, they underwent a total of 13 inpatient admissions, approximately twice per year, with each admission lasting 4 weeks, complemented by regular outpatient visits averaging three or more sessions per week. Throughout this period, both twins underwent a comprehensive rehabilitation program that was dynamically tailored to their respective functional levels at each stage of their clinical course.

The rehabilitation protocol was multifaceted, encompassing a wide spectrum of therapeutic modalities. Considering the pathophysiological vulnerabilities associated with DMD, all interventions were strictly maintained at a submaximal intensity to prevent muscle overuse, fatigue, and subsequent eccentric contraction-induced muscle destruction. Physical and occupational therapy formed the foundational components, structured as 30 min sessions twice daily for each, incorporating lower-extremity stretching, low-resistance strengthening to mitigate atrophy, and functional training. Therapeutic progression during functional training followed real-time capacity; for example, when independent standing balance deteriorated, the protocol shifted to targeted balance training and assisted standing exercises. While Twin A performed independent gait training during the first admission in May 2021, he also lost independent ambulation by the second admission in August 2021. Afterward, although their detailed GMFM-88 scores diverged, the core components of their functional training remained conceptually similar, as both had a non-ambulatory status requiring assisted standing and gait exercises. Aquatic therapy provided an additional avenue for low-resistance buoyancy-assisted exercise (30 min, 1–2 times per week). Robot-assisted gait training was further integrated as a core element of locomotor rehabilitation, employing three distinct categories of robotic systems: a treadmill-based tethered exoskeletal robot (Lokomat; Hocoma AG, Volketswil, Switzerland), an end-effector type robot (Morningwalk; CUREXO Inc., Seoul, Republic of Korea), and a torque-assisted overground wearable exoskeletal robot (Angellegs; Angel Robotics Co., Ltd., Seoul, Republic of Korea).

Concomitant medications, including corticosteroid regimens and gene therapy, were also maintained on an identical schedule for both twins. Specifically, both initiated delandistrogene moxeparvovec-rokl (Elevidys; Sarepta Therapeutics, Inc., Cambridge, MA, USA) on 9 June 2025, at another institution through a separate clinical trial, receiving the exact same dose concurrently.

**Figure 1:**
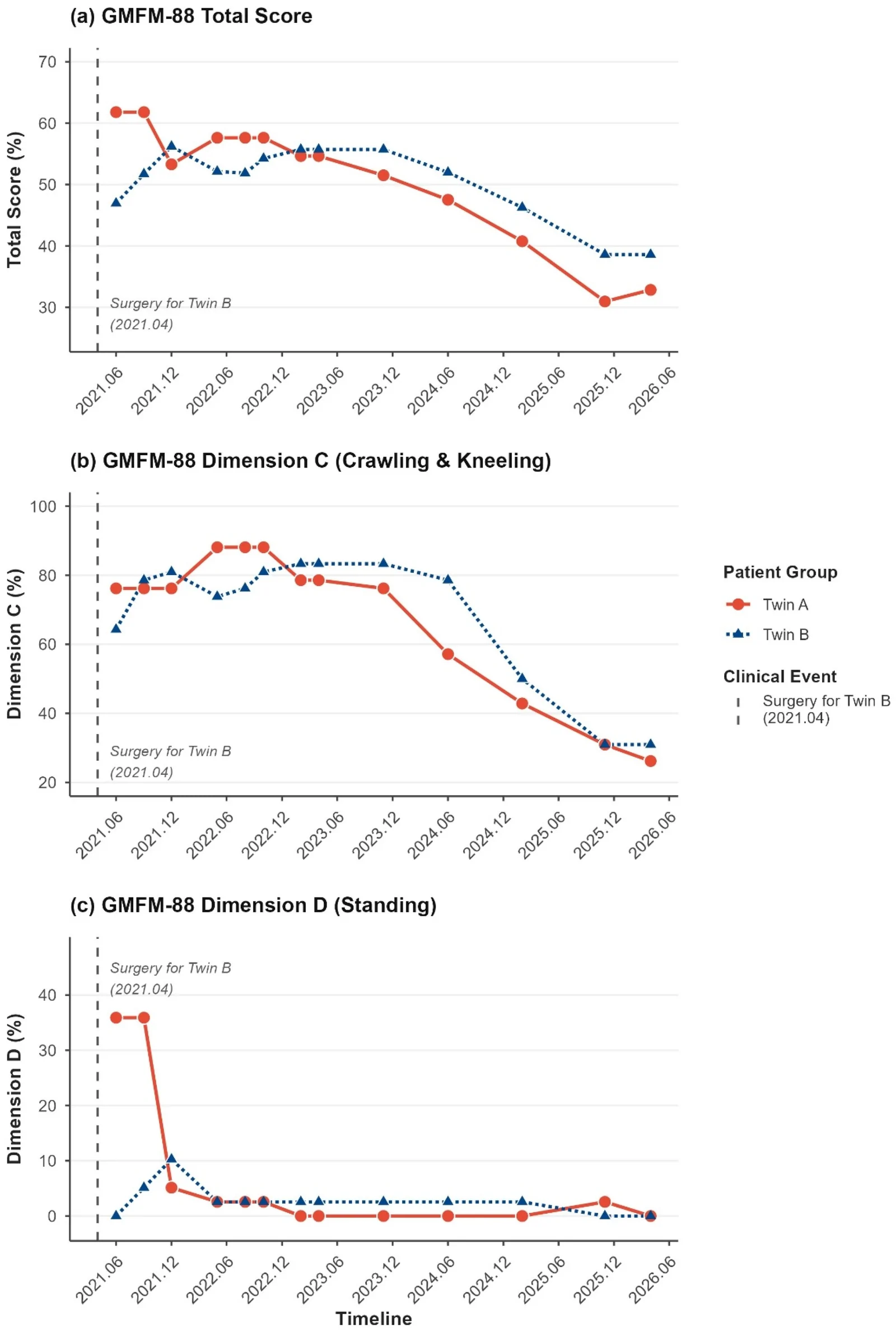
Longitudinal trajectory comparison between Twins A (red circles) and Twin B (blue triangles). Percentages (%) in panels (**a**–**c**) represent normalized scores calculated as the percentage of the maximum possible score achieved for each respective Gross Motor Function Measure-88 (GMFM-88) component. (**a**) GMFM-88 Total Score; (**b**) GMFM-88 Dimension C (Crawling & Kneeling); and (**c**) GMFM-88 Dimension D (Standing).

Throughout the treatment period, comprehensive longitudinal follow-up assessments were retrospectively obtained from medical records synchronized with each inpatient admission. This ensured identical assessment timing for both twins, as detailed in the time axes of Figure 1. As a retrospective review of routine clinical practice, assessor blinding was not performed. These evaluations included height and weight measurements, the Gross Motor Function Measure-88 (GMFM-88), and the Pediatric Evaluation of Disability Inventory—Computer Adaptive Test (PEDI-CAT). Although the twins were evaluated by different therapists over the study duration due to the retrospective nature of this study, all participating pediatric physical and occupational therapists completed identical institutional standardized training based on these tools upon employment. Nevertheless, the repeated measurements by different evaluators over the five-year period introduce a potential risk of inter-rater variability, which may affect the precise comparability of these functional metrics across different time points.

#### 2.2.1. Twin A (Non-Surgical Patient) Clinical Course

At his initial presentation in May 2021, Twin A displayed a mild equinus tendency but remained capable of independent ambulation for up to 5 min, with a baseline GMFM-88 total score of 61.8%. Despite conservative management utilizing hinged ankle-foot orthosis (AFO) with locking, his motor function progressively deteriorated. By his second admission in August 2021, he had lost independent ambulation and transitioned to a two-hand-supported gait. During his third admission in November 2021, given the rapid progression of the equinus deformity, therapeutic ultrasound was initiated to facilitate heel cord stretching.

He transitioned to a power wheelchair in September 2022. To address the progressive equinus contracture, serial casting was performed in November 2025, and a repeat course of serial casting is currently underway as of May 2026.

#### 2.2.2. Twin B (Surgical Patient) Clinical Course

Concurrent with a diagnostic biopsy on 14 April 2021, Twin B underwent bilateral minimally invasive ATL via three 1 cm heel cord incisions using a Beaver blade. Immediately after the surgery, a short-leg walking cast was applied in the neutral position, and the patient was encouraged to perform immediate full weight-bearing in the cast to prevent muscle disuse. The cast was removed five weeks later, followed by transition to a hinged AFO with locking. Prior to the surgery, he had developed a toe-walking gait 14 months prior to presentation and subsequently lost his ability to walk independently around March 2021. At his evaluation in April 2021, he was in the early non-ambulatory phase. Passive ankle dorsiflexion was −15° (right)/−10° (left) with the knee extended and 0°/0° at 90° flexion, indicating a gastrocnemius-dominant contracture. Medical Research Council grades were 4 for knee extensors/ankle plantarflexors and 3 for hip flexors/ankle dorsiflexors/great toe extensors. Immediate ATL was indicated to correct this early contracture while the proximal strength was preserved, optimizing alignment before complete muscle disuse. The evaluation 5 weeks after the procedure showed a temporary decline in his GMFM-88 total score (46.96%), which was lower than that of Twin A. With postoperative rehabilitation, he achieved stable, total-contact standing balance while holding a bar with the assistance of the hinged AFO with locking.

His gross motor scores gradually improved, and by his third admission in November 2021, his GMFM-88 total score increased to 56.22%, surpassing that of Twin A (53.7%). Although he transitioned to a power wheelchair at the same time as Twin A and remained unable to ambulate independently, his ankle dorsiflexion range of motion (ROM) remained well preserved throughout the follow-up period. Consequently, additional distressing interventions, such as serial casting, were avoided, and he maintained slightly higher final GMFM-88 total scores than Twin A following his third admission.

### 2.3. Quantitative Indicators and Longitudinal Data: GMFM-88 Trajectory

While Twin A initially demonstrated superior gross motor function, Twin B, who secured a stable ankle ROM via early surgery, exhibited a more stable, favorable GMFM-88 trajectory in the long term, as both transitioned through the non-ambulatory phase. Notably, during the late follow-up period spanning 2025 to 2026, Twin A’s gross motor function declined to 32.85%, whereas Twin B maintained a superior profile at 38.6% (Figure 1a). Among the five categories of GMFM-88, this divergence appeared to be driven by Dimensions C (Crawling & Kneeling) and D (Standing) trajectories. In Dimension C (Figure 1b), the initial trend was reversed in Twin B from February 2023 onward, outperforming Twin A by April 2026 (30.95% vs. 26.19%). Regarding Dimension D (Figure 1c), whereas Twin A experienced an abrupt and complete loss of standing function (0.00%) in February 2023, the residual standing capacity of Twin B was prolonged at 2.56% for an additional 2 years and 9 months before reaching 0.00% in November 2025.

### 2.4. QoL Metrics (KIDSCREEN)

To assess health-related QoL, the child self-reported KIDSCREEN-52 questionnaire was administered longitudinally between 2024 and 2026. This instrument evaluates 10 domains [5,6]: (1) physical well-being, (2) psychological well-being, (3) moods and emotions, (4) self-perception, (5) autonomy, (6) parent relation and home life, (7) social support and peers, (8) school environment, (9) social acceptance, and (10) financial resources. Raw scores were converted to standardized T-scores (mean = 50, SD = 10), with a 5-point change defined as the minimal clinically important difference (MCID). However, because the validity of this general threshold has not been fully established specifically for pediatric neuromuscular disorders, the changes in these longitudinal scores must be interpreted with caution.

T-score tracking showed synchronized baselines across most domains. Clinically significant changes exceeding the 5-point MCID occurred exclusively in Domains 2 (psychological well-being) and 7 (social support and peers) (Table 2). In Domain 2, Twin B (surgical) demonstrated an increase of 10.9 points, whereas Twin A (conservative) showed a minimal change (delta = 2.3). Conversely, in Domain 7, Twin A improved by 8.7 points, whereas Twin B showed no longitudinal change (delta = 0.0). In Domain 10, Twin A improved by 12.0 points, whereas Twin B showed no change (delta = 0.0). No other domains met the MCID threshold.

## 3. Discussion

This case report presents a retrospective comparative analysis of monozygotic twins with DMD who exhibited divergent clinical trajectories and QoL outcomes associated with the inclusion of ATL during the early stages of disease progression. This case has academic value as a well-controlled twin study that explores the potential benefits and critical considerations of orthopedic interventions in this population.

The clinical efficacy of orthopedic surgeries, specifically ATL, for equinus contracture in patients with DMD remains controversial, with ongoing debates regarding whether and when to operate [3,4,7,8,9]. According to Skalsky et al. and McDonald et al., the indiscriminate performance of ATL in patients who still retain ambulatory function can disrupt the unique compensatory mechanism achieved through toe-walking, paradoxically leading to the premature loss of independent ambulation. Consequently, the updated Birnkrant et al. guidelines do not advocate for a standardized surgical approach; instead, they emphasize a highly patient-specific strategy. Specifically, to consider surgical intervention, proximal muscle strength in the hips and knees must be sufficiently preserved, and the procedure must be meticulously timed during the transition phase before entering the fully non-ambulatory stage. Furthermore, the success of the intervention relies heavily on rigorous and structured postoperative rehabilitation, including physical therapy, occupational therapy, and robot-assisted gait training.

Twin B, in the present report, can be cautiously interpreted as a case where these rigorous criteria were relatively well satisfied. At the time of surgery, Twin B was transitioning to the non-ambulatory phase but still retained a degree of proximal muscle strength. Although he had transitioned to a non-ambulatory state 1 month prior to the evaluation, he presented with preserved proximal extensor strength and an equinus contracture primarily localized to the gastrocnemius rather than a rigid joint deformity. Immediately after surgery, he transitioned to a locked hinged AFO and underwent consistent inpatient and outpatient rehabilitation. Consequently, although the non-surgically treated twin (Twin A) initially demonstrated superior baseline GMFM-88 scores, the longitudinal evaluation revealed that Twin B maintained a more stable trajectory, eventually marginally surpassing Twin A’s GMFM-88 scores. This trend in the GMFM-88 total score appeared to be mainly driven by Dimension C (crawling and kneeling) and Dimension D (standing). The secured ankle ROM widened the base of support during standing, kneeling, and crawling, which likely allowed Twin B to maintain these dimensions relatively longer. This finding is supported by previous studies [10], which demonstrated that securing the ankle dorsiflexion ROM enhances standing, kneeling, and crawling abilities. These dimensions are hypothesized to have clinical relevance in the later stages of DMD, as the preservation of crawling, kneeling, and residual standing capacity might conceptually contribute to a patient’s ability to manage assisted transfers, maintain postural positioning, or participate in rehabilitation programs. Consequently, maintaining these abilities could potentially alleviate the caregiver burden and support psychological autonomy during the progressive physical decline.

This observation suggests that while surgical intervention cannot arrest the progressive decline in ambulatory functions in DMD, it may be associated with an attenuated rate of progressive motor deterioration when combined with stringent patient selection and thorough postoperative care. Given the simultaneous administration of multiple interventions, the specific, independent impact of the surgical procedure remains difficult to distinguish from that of the intensive rehabilitation program or other concurrent therapies. Nonetheless, from a theoretical standpoint, early ATL may have contributed by improving lower extremity alignment and achieving total foot-to-ground contact, which coincided with a less aggressive functional decline in Twin B.

Between 2024 and 2026, a longitudinal assessment of the child self-reported KIDSCREEN-52 revealed nuanced divergence between the twins. Whereas Twin B demonstrated a 10.9-point improvement in Domain 2 (psychological well-being), Twin A showed meaningful gains in Domain 7 (social support and peers; 8.7 points) and Domain 10 (financial resources; 12.0 points). These mixed results across different dimensions weaken any simplified narrative regarding the impact of orthopedic management on health-related QoL, and these findings should be presented strictly as exploratory observations. While it is plausible that Twin B’s preserved ankle ROM coincided with a more comfortable rehabilitation experience, this trend cannot be directly attributed to a therapeutic effect of the surgical intervention. QoL metrics are highly complex and multifactorial; these divergent changes likely reflect a combination of distinct individual coping mechanisms, evolving peer dynamics, and broader family and social support factors, rather than the surgical intervention alone.

Taken together, this case demonstrates that surgical intervention should not be categorically dismissed during the management of equinus contracture in patients with DMD, nor should the decision-making process be strictly limited to immediate gait parameters. Although determining whether to operate and identifying the optimal surgical timing remain highly controversial, the divergent outcomes observed here suggest that the choice to perform surgery coincided with differences in the clinical trajectory, whereas the longitudinal QoL profiles remained highly mixed and inconclusive, preventing any direct causal attribution. It must be emphasized that the success of such surgical interventions is not absolute on its own; indeed, ATL can induce a transient phase of acute functional deterioration immediately after surgery. However, as observed in Twin B, patients may overcome this initial decline when surgical intervention is coupled with structured post-operative rehabilitation. Given that this is based on a single-pair twin case, these findings must be interpreted as suggestive. Nonetheless, rather than offering immediate clinical directives, this single-pair observation highlights the potential value of expanding the evaluative focus in future research beyond short-term ambulation metrics to comprehensively consider long-term functional resilience and late-stage well-being within a multi-disciplinary care regimen.

This case report had several limitations that merit consideration. First, because this study involves a single twin pair subjected to multiple co-interventions, the findings support only a longitudinal association, not a direct causal link, and cannot be generalized to the wider DMD population. Second, although both twins initiated delandistrogene moxeparvovec-rokl concurrently on an identical schedule and at the same dose, the potential confounding influence of gene therapy on longitudinal functional and QoL trajectories cannot be entirely excluded. Similarly, the potential confounding effects of concurrent rehabilitation modalities cannot be entirely excluded. Nonetheless, the longitudinal divergence in functional and psychosocial outcomes documented before gene therapy initiation lends credibility to the interpretation that the observed inter-twin differences are primarily attributable to differential surgical management. Future longitudinal studies comparing pre- and post-gene-therapy trajectories are warranted to clarify these interactions. Third, the divergent improvements across different KIDSCREEN-52 domains underscore the difficulty in isolating surgical effects on psychosocial outcomes. Because subjective QoL reporting is deeply influenced by unmeasured familial, environmental, and social variables, a direct causal link to the orthopedic intervention cannot be confirmed, necessitating strict caution when generalizing these findings.

## 4. Conclusions

Although ATL does not arrest the progression of DMD, this twin case serves as a hypothesis-generating observation, highlighting a potential longitudinal association between early surgery and attenuated progressive motor decline within the context of a multi-disciplinary care regimen, while the psychosocial outcomes remain highly variable and exploratory in nature. While the single-pair design inherently limits direct generalization to established clinical guidelines, these findings nonetheless raise the possibility that evaluating such orthopedic interventions may benefit from looking beyond immediate gait metrics toward broader, long-term functional trends. Crucially, any potential long-term benefits remain speculative, and the relative contributions of the surgical intervention, the intensive rehabilitation program, or their potential synergy remain to be elucidated in future studies.

## Figures and Tables

**Table 1:** Comparison of clinical milestones and interventions between twins.

	First Symptom	Ambulation Loss	Diagnosis (Method)	Surgery	First Admission for Rehabilitation	First Orthosis Application (Type)	Power Wheelchair Transition	Gene Therapy
Twin A	May 2020 (toe gait)	August 2021	June 2021 (NGS)	None	May 2021	June 2021 (hinged AFO)	September 2022	June 2025
Twin B	January 2020 (toe gait)	March 2021	April 2021 (muscle biopsy, NGS)	April 2021	May 2021	May 2021 (hinged AFO)	September 2022	June 2025

AFO, ankle-foot orthosis; NGS, next-generation sequencing.

**Table 2:** Longitudinal profiles and inter-twin differences in KIDSCREEN-52 T-scores.

	Twin A			Twin B			
Domain	2024	2026	ΔT-Score	2024	2026	ΔT-Score	Twin B—Twin A
**1. Physical well-being**	38.4	47.6	9.2	38.4	49.5	11.1	+1.9
**2. Psychological well-being**	58.2	60.5	2.3	60.5	71.4	10.9	+8.6 *
**3. Moods and emotions**	71.1	82.3	11.2	71.1	82.3	11.2	0.0
**4. Self-Perception**	71.8	78.2	6.4	67.2	78.2	11.0	+4.6
**5. Autonomy**	56.7	61.4	5.7	56.7	61.4	4.7	0.0
**6. Parent relation and home life**	76.1	76.1	0.0	76.1	76.1	0.0	0.0
**7. Social support and peers**	46.8	55.5	8.7	46.8	46.8	0.0	−8.7 *
**8. School environment**	41.4	50.6	9.2	39.6	48.7	9.1	−0.1
**9. Social acceptance**	71.4	71.4	0.0	71.4	71.4	0.0	0.0
**10. Financial resources**	60.3	72.3	12.0	60.3	60.3	0.0	−12.0 *

ΔT-score (2024–2026); * values exceeding 5 points, which is the minimal clinically important difference (MCID).

## Data Availability

The data presented in this study are available on request from the corresponding author. The data are not publicly available due to individual privacy.

## Abbreviations

The following abbreviations are used in this manuscript:

AFO	ankle-foot orthosis
ATL	Achilles tendon lengthening
DMD	Duchenne muscular dystrophy
GMFM-88	Gross Motor Function Measure-88
MCID	minimal clinically important difference
MEP	maximal expiratory pressure
MIP	maximal inspiratory pressure
NGS	next-generation sequencing
PCF	peak cough flow
PEDI-CAT	Pediatric Evaluation of Disability Inventory—Computer Adaptive Test
QoL	quality of life
ROM	range of motion
VC	vital capacity

## References

[1] Bushby K., Finkel R., Birnkrant D.J., Case L.E., Clemens P.R., Cripe L., Kaul A., Kinnett K., McDonald C., Pandya S. (2010). Diagnosis and management of Duchenne muscular dystrophy, part 1: Diagnosis, and pharmacological and psychosocial management. Lancet Neurol..

[2] de Souza M.A., Figueiredo M.M.L., de Baptista C.R.d.J.A., Aldaves R.D., Mattiello-Sverzut A.C. (2016). Beneficial effects of ankle–foot orthosis daytime use on the gait of Duchenne muscular dystrophy patients. Clin. Biomech..

[3] Birnkrant D.J., Bushby K., Bann C.M., Alman B.A., Apkon S.D., Blackwell A., Case L.E., Cripe L., Hadjiyannakis S., Olson A.K. (2018). Diagnosis and management of Duchenne muscular dystrophy, part 2: Respiratory, cardiac, bone health, and orthopaedic management. Lancet Neurol..

[4] Skalsky A.J., McDonald C.M. (2012). Prevention and management of limb contractures in neuromuscular diseases. Phys. Med. Rehabil. Clin. N. Am..

[5] Hong S.D., Yang J.W., Jang W.S., Byun H., Lee M.S., Kim H.S., Oh M.-Y., Kim J.-H. (2007). The KIDSCREEN-52 quality of life measure for children and adolescents (KIDSCREEN-52-HRQOL): Reliability and validity of the Korean version. J. Korean Med. Sci..

[6] Ravens-Sieberer U., Gosch A., Rajmil L., Erhart M., Bruil J., Duer W., Auquier P., Power M., Abel T., Czemy L. (2005). KIDSCREEN-52 quality-of-life measure for children and adolescents. Expert Rev. Pharmacoecon. Outcomes Res..

[7] Lejman J., Pytlak M., Danielewicz A., Rutz E., Latalski M., Lejman M. (2026). Molecular Insights and Orthopedic Management in Muscular Dystrophies: A Comprehensive Review. Int. J. Mol. Sci..

[8] Forst J., Forst R. (2012). Surgical treatment of Duchenne muscular dystrophy patients in Germany: The present situation. Acta Myol..

[9] Apkon S.D., Alman B., Birnkrant D.J., Fitch R., Lark R., Mackenzie W., Weidner N., Sussman M. (2018). Orthopedic and surgical management of the patient with Duchenne muscular dystrophy. Pediatrics.

[10] Moses M.O., Onuegbu N.F., Deku P.D.-G., Nyarko M.A., Owusu L.B., Emikpe A.O., John E.B., Soangra R., Yifieyeh A.C., Titiloye N.A. (2025). Joint Angular Kinematics and Gross Motor Function in Typically Developing Healthy Children. Children.

